# Using Sniffing Behavior to Differentiate True Negative from False Negative Responses in Trained Scent-Detection Dogs

**DOI:** 10.1093/chemse/bju045

**Published:** 2014-09-11

**Authors:** Astrid Concha, Daniel S. Mills, Alexandre Feugier, Helen Zulch, Claire Guest, Rob Harris, Thomas W. Pike

**Affiliations:** ^1^School of Life Sciences, University of Lincoln, Joseph Banks Building, Lincoln LN6 7DL, UK,; ^2^Royal Canin SAS, Avenue de la petite Camargue, Aimargues F-30470, France and; ^3^Medical Detection Dogs, 3 Millfield, Greenway Business Park, Great Horwood, Milton Keynes MK17 0NP, UK

**Keywords:** detection dogs, false negative, false positive, sniffing behavior, target odor

## Abstract

False negatives are recorded in every chemical detection system, but when animals are used as a scent detector, some false negatives can arise as a result of a failure in the link between detection and the trained alert response, or a failure of the handler to identify the positive alert. A false negative response can be critical in certain scenarios, such as searching for a live person or detecting explosives. In this study, we investigated whether the nature of sniffing behavior in trained detection dogs during a controlled scent-detection task differs in response to true positives, true negatives, false positives, and false negatives. A total of 200 videos of 10 working detection dogs were pseudorandomly selected and analyzed frame by frame to quantify sniffing duration and the number of sniffing episodes recorded in a Go/No-Go single scent-detection task using an eight-choice test apparatus. We found that the sniffing duration of true negatives is significantly shorter than false negatives, true positives, and false positives. Furthermore, dogs only ever performed one sniffing episode towards true negatives, but two sniffing episodes commonly occurred in the other situations. These results demonstrate how the nature of sniffing can be used to more effectively assess odor detection by dogs used as biological detection devices.

## Introduction

Chemical detection systems are widely used to recognize the presence of a particular substance or identify low concentration of volatile compounds and hazardous gases by mimicking an animal’s sense of smell (Glatz and Bailey-Hill 2011; Oh *et al.* 2011; Lee *et al.* 2012). Although recent advances have improved the precision and efficacy of these detection technologies, these are still imperfect (Dacres *et al.* 2011; Zhang *et al.* 2013), and animals continue to appear more sensitive than man-made systems (Shelby *et al.* 2006; Macias *et al.* 2010; Weber *et al.* 2011; Bomers *et al.* 2012; Horvath *et al.* 2013), in addition to having the advantage of being a more dynamic system allowing quick detection over a large search area (Calbk *et al.* 2008). However, regardless of the nature of the detection system both false positive (where the system detects the target as present when it is absent) and false negative (where the target is present but the system fails to detect it) errors occur in these and in every detection system. The proportion of these errors occurring in a working scenario is a measure of the accuracy of the detection system. These errors may occur as a result of observation error (Mudford *et al.* 2009) as if the operator is not able to recognize and interpret the results obtained by the device, then the reliability of the detection system is affected. This is particularly a risk when animals are used as biological detector devices, because detection performance is assessed by handlers (Townsend 2003; Habib 2007). The dog (*Canis familiaris*) is the most widely employed scent-detector device for civilian and military purposes (Brook and Koehler 2003; Osterkamp 2011; Rooney *et al.* 2013), and these errors are well documented; both a failure to respond correctly to the presence or absence of the target odor (Bach and McLean 2003), and false negative and false positive results recorded due to handler error (Lasseter *et al.* 2003; Wasser *et al.* 2004; Lit *et al.* 2011). It is, therefore, important to investigate factors which may help to differentiate where the error may lie. In the case of an apparent false negative, there may be a failure of the dog to detect the presence of the target odor, or a failure of the handler to recognize that the dog has detected the odor. A reduction in false negatives is particularly valuable in situations, such as when a detection dog is used for searching for a live person, detecting explosives, or identifying perpetrators of a crime.

Sniffing behavior is obviously important in the detection and discrimination of odors (Sobel *et al.* 2000; Verhagen *et al.* 2007). This is actively controlled during investigatory behavior and rapidly modulated in response to sensory input to optimize the transport of volatile compounds to the olfactory epithelium and thus for olfactory processing (Kepecs *et al.* 2007; Wachowiak 2011). However, whether the sniffing behavior in dogs is modified by the presence of the target odor in a scent-detection task (and so could be used as an indicator of false alerts, both negative and positive) has not been investigated. We, therefore, analyzed whether the sniffing behavior of detection dogs differs according to the olfactory detection parameters noted (true positives, true negatives, false positives, and false negatives) during a scent-detection task. It was hypothesized that when the target odor is not present, then sniffing duration will be shorter (true negatives and possibly some false positives), and that dogs will be more likely to reinvestigate marginal signals they perceive as inconclusive before issuing a response (false negatives and some false positives).

## Materials and methods

### Ethics statement

This research was approved by the School of Life Sciences Ethics Committee at the University of Lincoln, United Kingdom. All dogs were trained according to the ethical guidelines established by the charity Medical Detection Dogs (UK charity registration number 1124533).

### Subjects

This study involved 10 detection dogs, 4 females and 6 males, ranging in age from 30 to 138 months (mean ± standard deviation [SD]: 64.3±38.52 month), with body weights from 10.5 to 24.0kg (mean ± SD: 19.24±3.97kg), and of the following breeds: Cocker Spaniel (*n* = 3), Labrador Retriever (*n* = 3), Border Collie (*n* = 2), and English Springer Spaniel (*n* = 2).

### Odor sample preparation and training procedure

The dogs were trained to detect solutions of pentyl acetate (amyl acetate, CAS 628-63-7; ≥99% Sigma Aldrich, W504009) diluted in mineral oil (Sigma Aldrich, M8410) at different concentrations. A simple dilution from a stock solution of 1:1000 pentyl acetate:mineral oil (0.5mL amyl acetate in 499.5mL mineral oil) was used to prepare samples with concentrations above 1:1000000. One to three steps of 10-fold serial dilutions of this stock solution were used to maximize the consistency of preparation of the target odor concentrations below 1:1000000. One milliliter of the target concentration was required for each session and placed in a sterile 60mL screw-top polypropylene container (4cm diameter, item number 360103PP; Wheaton). Seven controls, each made up of 1mL of mineral oil, were deposited in identical sterile containers. Each set of containers were used only in one session and subsequently discarded. The target and control odor containers were opened and set up on an eight-choice carousel, similar to the circular stainless steel odor presentation system which has been used in other studies (Fjellanger *et al.* 2002; Sargisson and McLean 2010).

Three concentrations of pentyl acetate were presented daily for each dog in a training session. The target concentrations were presented to the dogs in a systematic lowering of concentration. The rate of decrease in concentrations was 50% below the level detected earlier by the dog, based on its individual proportion of true positives obtained by concentration. During the detection training, the dogs were exposed to a range of concentrations, determined by each dog’s ability from 1:10000 to 1:1500000000.

The dogs worked in an indoor room (~20 °C and 51% humidity) at the charity Medical Detection Dogs. They worked with the same handler (R.H.) throughout, and had been trained using the technique of forward chaining with a clicker and a food reward (Educ Royal Canin®). Dogs were paired on the basis of their performance in detecting similar concentrations, and each pair worked the same set of samples (target odor and controls). The order in which dogs worked (first or second) was counterbalanced during each session over different target concentrations. Sessions involved runs and passes. A run began when the target changed its position on the carousel (e.g., changed from arm 3 to 8), whereas a pass was when the dog searched the individual carousel arms 1 to 8.

The position of the target in the carousel was determined randomly for each run using custom-made computer software, and the handler was blind regarding the position of the target in the carousel and the target concentration tested. The target and controls were placed on the carousel by the same researcher (A.C.), whereas the dog and handler were in a separate room. The time between the placement of the target and controls in the carousel and the beginning of the search was between 5 and 10min, giving time for the odors to stabilize in the headspace of their containers.

**Figure 1:**
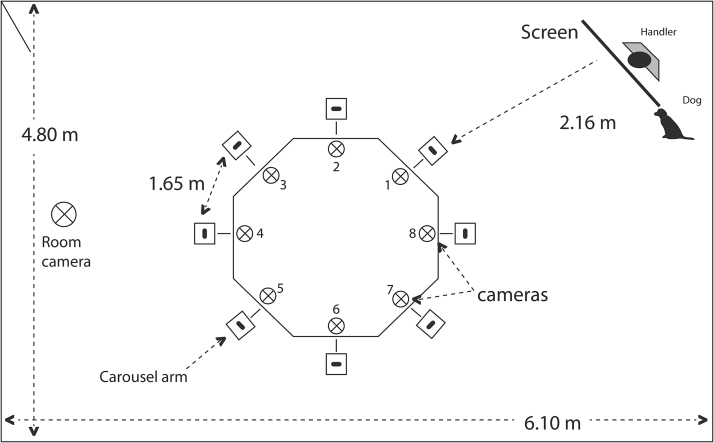
Schematic ilustration of the room layout. At the start of a session, the handler stood behind a screen and the dog was positioned next to him. The screen had a one way mirrored window at a height which made it possible for the handler to observe the dog, and was located 2.16 m from the carousel arm number 1. The handler remained behind the screen when dogs started searching the individual carousel arms circling either clockwise (from arm 8 to 1) or counterclockwise (from 1 to 8); the handler remained behind the screen during the search. The dogs were videorecorded via a ceiling-mounted camera and small individual cameras fixed on each carousel arm.

The handler and the dog entered the room together and left the room between each run, but remained inside between passes. The experimenter left the room when the handler entered. Once inside the room, the handler stood behind a screen (with a one way mirror window at a height which made it possible for the handler to observe the dog without being seeing by it) and the dog was positioned next to the handler (Figure 1). Each session consisted of two runs per concentration and two passes per run. However, a third pass was allowed when the dog did not appear to search for the position of a target on the earlier two passes. The dog could start every pass from an initial position (next to the handler) or carry on searching for a consecutive second pass. The handler, who remained behind the screen, gave a verbal command to the dog to start the search. Dogs sniffed the individual carousel arms circling either clockwise or counterclockwise without the assistance of the handler who remained behind the screen. When a dog showed the trained alert response (“sit”) at a position on the carousel, the handler confirmed the position through the computer program; if the indication of the dog was correct (true positive) it was clicked, the dog left the carousel position and returned to the initial position (next to the handler) to be rewarded by the handler with three pieces of Educ Royal Canin®. If a false positive response was given, the behavior of the dogs was not reinforced.

A new clean set of arms was placed on the carousel in each session. In addition, the carousel was cleaned with distilled water, the arms boiled, and the test room vacuumed every day to decrease the possibility of contamination, in accordance with the normal procedure of the charity.

### Data analysis

The olfactory detection performance of the dog was defined in accordance with Signal-detection theory (Fjellanger *et al.* 2002;Macmillan and Creelman 2005; Furton *et al.* 2010) as follows: 1) True positive: the dog indicates the target odor in the manner in which it was trained (“sit” response), 2) False positive: the dog alerts to a nontarget position (control), 3) False negative: the dog fails to exhibit the trained alert in the presence of the target odor, and 4) True negative: the dog does not alert in the absence of the target odor.

The dogs were videotaped during every training session via a ceiling-mounted video camera (Sentient Wired® outdoor camera model N94FY), and small individual cameras (8 Channel DVR, RF2421 8ch H.263 Model 2005XA B/W EXVIEW 3.6mm CCIR [Pal]) fixed on each carousel arm (Figure 1). A total of 200 videos were pseudorandomly selected from the videos available of the detection training (Excel® random number generation), such that 20 videos were chosen for each dog including five of each of the four response types, and including a range of target concentration, from 1:700000 to 1:1500000 (pentyl acetate:mineral oil). Frames from the selected videos (with a frame rate of 25 fps) were converted to individual JPEG images using Free Studio 3 (version 5.0.28), and used to quantify both sniffing duration (s) and the number of sniffing episodes (sniffs over the odor sample). The onset of a sniffing episode was defined from when the dog’s nose was put over the hole of the carousel arm, and the end point was when the dog’s nose moved away from it.

The dog’s response type was confirmed by assessing agreement with a blind independent rater for both sniffing duration and number sniffing episodes. There was significant level of interobserver agreement between the two raters for sniffing duration for both the first (*r* = 0.721, *n* = 20, *P* < 0.001) and second episodes (*r* = 0.923, *n* = 20, *P* < 0.05).

### Statistical analysis

All analyses were conducted in R.2.15.2 (http://www.r-project.org/). To determine whether sniffing duration before a choice is made differed as a function of response choice (true positive, true negative, false positive, and false negative), we used a general linear mixed model (implemented using the lmer function of the lme4 package; Pinheiro and Bates 2000) with dog identity as a random effect. We log10-transformed the duration of sniffing data prior to analysis to ensure normally distributed residuals in the model. Tukey’s honest significant differences test (using the glth function of the multcomp package) was used to compare between levels of response choice. Differences between response choices in the number of sniffing episodes were tested using a generalized linear mixed model (using the glmer function of the lme4 package) with a binomial error distribution and dog identity as a random effect. For those sequences in which dogs performed two sniffing episodes, the difference in the log10-transformed duration of sniffing between the first and second episodes was tested using a general linear model with dog identity and episode as random effects. In all cases, statistical significance was determined by comparing full models to models lacking the independent variable using likehood ratio tests (Crawley 2005). Pearson’s correlations were used to determine interobserver agreement between two independent raters for measuring of sniffing duration and counting sniffing episodes (Multon 2010). Results were considered statistically significant if *P* < 0.05.

## Results

The sniffing duration of dogs during the scent-detection task differed significantly between the four olfactory response choices (*F*_3,196_ = 13.89, *P* < 0.001). In particular, the dogs spent significantly less time sniffing true negative samples in the first episode than true positives, false positives, and false negatives (all Tukey-corrected *P* < 0.001, Table 1). Similarly the sniffing duration of false negatives was significantly shorter than true positives in the first episode (Tukey-corrected *P* < 0.05).

**Table 1:** Sniffing duration for the first and second sniffing episode as a function of the olfactory response choice

Olfactory detection parameter	First sniffing episode (s) (mean ± SD)	Second sniffing episode (s) (mean ± SD)
True positive	0.498±0.239^b,c^	0.257±0.129
True negative	0.268±0.118^a^	—
False positives	0.468±0.223^b^	0.288±0.175
False negative	0.408±0.714^b,d^	0.224±0.096

Olfactory parameters with different superscript letter differ significantly from one another (^a,b^ *P* < 0.001; ^c,d^ *P* < 0.05) during the first sniffing episode. *N* = 50 for each parameter.

The presence of a second sniffing episode was observed during false positives, true positives, and false negatives, but not during true negative samples (χ^2^_3_ = 82.79, *P* < 0.001). Overall, the mean sniffing duration of the first sniffing episode across the olfactory response choices was significantly longer than the second episode (*F*_1,112_ = 30.31, *P* < 0.001, Table 1).

## Discussion

In detection dogs, the accuracy of the detection depends on both the dog’s olfactory capability to identify the target odor and the interpretation of the dog’s behavior by a handler. Earlier studies in detection dogs have not directly analyzed the relationship between sniffing behavior and accuracy of odor discrimination in detection tasks, concentrating instead on the total duration of the search (Thesen *et al.* 1993; Jezierski *et al.* 2008). Our results indicate that sniffing behavior can be used alongside the trained alert response to more effectively assess detection. Specifically, we found that the sniffing duration of detection dogs used in this study is shortest when the target odor is not present and the dogs indicate this by not offering an alert response (true negative), and the dogs only ever performed one sniffing episode towards these samples compared with the other responses (true positive, false positive, and false negative). In particular, samples that resulted in true positive, false positive, and false negative decisions were sniffed for approximately twice the amount of time of true negatives. In other words, the detection dogs used in this study sniffed for twice the amount of time when the target odor was present or when it was indicated as present on a negative sample.

The shorter sniffing duration shown during true negative responses indicates that initial encoding of the presence–absence of a stimulus is rapid (Wesson *et al.* 2009) with discrimination determined with a single sniff (Mainland and Sobel 2006). This is comparable to observations in rodents where the sniffing duration for odor discrimination lasted between 0.15 and 0.20 s in a similar detection task (Uchida and Mainen 2003; Abraham *et al.* 2004; Kepecs *et al.* 2007). Wesson *et al.* (2008) demonstrated that the time between the first sniff and the olfactory receptor input reaching the olfactory bulb is 0.1–0.15 s, leaving only 0.05–0.1 s for the central processing and instigation of the discriminative behavioral response. Prolonged sniffing does not seem to be necessary for the detection when the target odor is absent (true negative indications). Similar results have been described by Slotnick (2007) in rodents where longer sniffing duration was evident when determination that the target odor was present occurred. This has been interpreted as indicating that the cognitive processing for detecting whether the target odor is present or not occurs separately from the identification and recognition of the target odor. False positive responses can arise from the identification of background compounds similar to the target odor (Kurz *et al.* 1996) and the presence of extraneous odors (Bach and McLean 2003). Thus, the longer sniffing duration found in our study towards true positive, false positive, and false negative responses might reflect the engagement of higher-order pathways associated with the recognition of the odor itself.

The analysis of sniffing behavior frame by frame has been used earlier to evaluate nostril laterality in untrained dogs during the investigation of cotton swabs impregnated with different odorants (Siniscalchi *et al.* 2011). This technique allows a more detailed evaluation of some of the characteristics of sniffing behavior. The high level of agreement between two independent observers using this approach also shows that it is highly reliable method for objectively quantifying behavioral occurrences in extremely short periods of time. However, the application of this method for measuring sniffing duration simultaneously with the dog searching for the target odor is perhaps more limited.

Overall, the findings from this study provide evidence that sniffing behavior can be used to effectively assess olfactory alert performance in detection dogs beyond the trained alert response and was particularly valuable in differentiating true from false negative responses: an area where the consequences of error may be serious in real search scenarios such as mine and explosive detection or the search for a live person. Other aspects of dogs’ behavior regarding olfactory detection and the alert response should be investigated to identify and standardize parameters to assess dogs’ alert responses regardless of the target odor or the working situation. Future work is ongoing to further investigate the generality of the findings reported here and develop technology to evaluate sniffing behavior in real time during search tasks under field conditions.

## Funding

This work was supported by Royal Canin SAS (grant name Nutritional factors and olfactory performance in dog).

## Conflict of interest

Royal Canin SAS supported the study financially; they approved the experimental design, financially supported Astrid Concha’s PhD candidature, and provided the food and Educ® food treats for the dogs used in this research. They did not have any influence over the study conception, design or interpretation, or the decision to publish the data. A.F. is employed by Royal Canin SAS. The authors neither have patent or stock ownership which would affect this research or publication nor have any membership of a company board of directors, membership of an advisory board or committee for a company, or consultancy for receipt of speaker's fees from a company.

## References

[CIT0001] Abraham NM Spors H Carleton A Margrie TW Kuner T Schaefer AT. 2004. Maintaining accuracy at the expense of speed: stimulus similarity defines odor discrimination time in mice. Neuron. 44:865–876 15572116 10.1016/j.neuron.2004.11.017

[CIT0002] Bach H McLean I. 2003. Remote explosive scent tracing (REST), genuine or a paper tiger? J Mine Action. 7:75–82

[CIT0003] Bomers MK van Agtmael MA Luik H van Veen MC Vandenbroucke-Grauls CM Smulders YM. 2012. Using a dog’s superior olfactory sensitivity to identify *Clostridium difficile* in stools and patients: proof of principle study. BMJ. 345:e7396 23241268 10.1136/bmj.e7396 PMC3675697

[CIT0004] Brook SE Koehler PG. 2003. Ability of canine termite detectors to locate alive termites and discriminate them from nontermite material. J Econ Entomol. 96:1259–1266 14503599 10.1093/jee/96.4.1259

[CIT0005] Calbk ME Sagebiel JC Heaton JS Valentin C. 2008. Olfaction-based detection distance: a quantitative analysis of how far away dogs recognize tortoise odor and follow it to source. Sensors. 8:2208–2222 10.3390/s8042208 PMC3673414 27879818

[CIT0006] Crawley MJ. 2005. Statistics: an introduction using R. 1st ed. Chichester (UK): John Wiley & Sons Ltd. p. 103–124

[CIT0007] Dacres H Wang J Leitch V Horne I Anderson AR Trowell SC. 2011. Greatly enhanced detection of a volatile ligand at femtomolar levels using bioluminescence resonance energy transfer (BRET). Biosens Bioelectron. 29:119–124 21873043 10.1016/j.bios.2011.08.004

[CIT0008] Fjellanger R Andersen EK McLean IG. 2002. A training program for Filter-Search Mine Detection Dogs. Inter J Comp Psych. 15:278–287

[CIT0009] Furton K Greb J Holness H. 2010. The scientific working group on dog and orthogonal detector guidelines (SWGDOG). Miami (FL): Florida International University. Available from: http://swgdog.fiu.edu/approved-guidelines/sc1_terminology_abcdefghijk.pdf

[CIT0010] Glatz R Bailey-Hill K. 2011. Mimicking nature’s noses: from receptor deorphaning to olfactory biosensing. Prog Neurobiol. 93:270–296 21130137 10.1016/j.pneurobio.2010.11.004

[CIT0011] Habib M. 2007. Humanitarian demining: reality and the challenge of technology—the state of the arts. Int J Adv Robot Syst. 4:151–172

[CIT0012] Horvath G Andersson H Nemes S. 2013. Cancer odor in the blood of ovarian cancer patients: a retrospective study of detection by dogs during treatment, 3 and 6 months afterward. BMC Cancer. 13:396 23978091 10.1186/1471-2407-13-396 PMC3765942

[CIT0013] Jezierski T Walczak M Gorecka A. 2008. Information-seeking behaviour of sniffer dogs during match-to-sample training in the scent lineup. Pol Psychol Bull. 39:71–80

[CIT0014] Kepecs A Uchida N Mainen ZF. 2007. Rapid and precise control of sniffing during olfactory discrimination in rats. J Neurophysiol. 98:205–213 17460109 10.1152/jn.00071.2007

[CIT0015] Kurz ME Schultz S Griffith J Broadus K Sparks J Dabdoub G Brock J. 1996. Effect of background interference on accelerant detection by canines. J Forensic Sci. 41:868–873 8789850

[CIT0016] Lasseter AE Jacobi KP Farley R Hensel L. 2003. Cadaver dog and handler team capabilities in the recovery of buried human remains in the southeastern United States. J Forensic Sci. 48:617–621 12762533

[CIT0017] Lee SH Kwon OS Song HS Park SJ Sung JH Jang J Park TH. 2012. Mimicking the human smell sensing mechanism with an artificial nose platform. Biomaterials. 33:1722–1729 22153868 10.1016/j.biomaterials.2011.11.044

[CIT0018] Lit L Schweitzer JB Oberbauer AM. 2011. Handler beliefs affect scent detection dog outcomes. Anim Cogn. 14:387–394 21225441 10.1007/s10071-010-0373-2 PMC3078300

[CIT0019] Macias MS Guerra-Diaz P Almirall JR Furton KG. 2010. Detection of piperonal emitted from polymer controlled odor mimic permeation systems utilizing Canis familiaris and solid phase microextraction-ion mobility spectrometry. Forensic Sci Int. 195:132–138 20044224 10.1016/j.forsciint.2009.12.006

[CIT0020] Macmillan N Creelman C. 2005. Basic detection theory and one-interval designs. In: Macmillan N Creelman C, editors. Detection theory: a user’s guide. 2nd ed. New York: Laurence Erlbaum Associates. p. 3–25

[CIT0021] Mainland J Sobel N. 2006. The sniff is part of the olfactory percept. Chem Senses. 31:181–196 16339268 10.1093/chemse/bjj012

[CIT0022] Mudford OC Martin NT Hui JK Taylor SA. 2009. Assessing observer accuracy in continuous recording of rate and duration: three algorithms compared. J Appl Behav Anal. 42:527–539 20190916 10.1901/jaba.2009.42-527 PMC2741073

[CIT0023] Multon K. 2010. Interrater reliability. In: Salkind NJ, editor. Encyclopedia of research design 1st ed. Thousand Oaks (CA): Sage publications. p. 627–629

[CIT0024] Oh EH Song HS Park TH. 2011. Recent advances in electronic and bioelectronics noses and their biomedical applications. Enzyme Microb Tech. 48:427–437 10.1016/j.enzmictec.2011.04.003 22113013

[CIT0025] Osterkamp T. 2011. K9 water searches: scent and scent transport considerations. J Forensic Sci. 56:907–912 21480897 10.1111/j.1556-4029.2011.01773.x

[CIT0026] Pinheiro J Bates D. 2000. Linear mixed-effects models: Basic concepts and examples. In: Mixed-effects models in S and S-Plus. 1st ed. New York: Springer Verlag. p. 3–52

[CIT0027] Rooney NJ Morant S Guest C. 2013. Investigation into the value of trained glycaemia alert dogs to clients with type I diabetes. PLoS One. 8:e69921 23950905 10.1371/journal.pone.0069921 PMC3737201

[CIT0028] Sargisson RJ McLean IG. 2010. The effect of reinforcement rate variations on hits and false alarms in remote explosive scent tracing with dogs. J ERW Mine Detect. 14.3: 64–68

[CIT0029] Shelby RA Myers LJ Schrader KK Klesius PH. 2006. Detection of off-flavour in channel catfish (*Ictalurus punctatus* Rafinesque) fillets by trained dogs. Aquacult Res. 37:299–301

[CIT0030] Siniscalchi M Sasso R Pepe AM Dimateo S Vallortigara G Quaranta A. 2011. Sniffing with the right nostril: lateralization of response to odour stimuli by dogs. Anim Behav. 82:398–404

[CIT0031] Slotnick B. 2007. Odor-sampling time of mice under different conditions. Chem Senses. 32:445–454 17426048 10.1093/chemse/bjm013

[CIT0032] Sobel N Khan RM Hartley CA Sullivan EV Gabrieli JD. 2000. Sniffing longer rather than stronger to maintain olfactory detection threshold. Chem Senses. 25:1–8 10667988 10.1093/chemse/25.1.1

[CIT0033] Thesen A Steen JB Dovich KB. 1993. Behavior of dogs during olfactory tracking. J Exp Biol. 180:247–251 8371085 10.1242/jeb.180.1.247

[CIT0034] Townsend J. 2003. Pigs: A demining tool of the future? J Mine Action. 7:43–46

[CIT0035] Uchida N Mainen ZF. 2003. Speed and accuracy of olfactory discrimination in the rat. Nat Neurosci. 6:1224–1229 14566341 10.1038/nn1142

[CIT0036] Verhagen JV Wesson DW Netoff TI White JA Wachowiak M. 2007. Sniffing controls an adaptive filter of sensory input to the olfactory bulb. Nat Neurosci. 10:631–639 17450136 10.1038/nn1892

[CIT0037] Wasser SK Devenport B Ramage ER Hunt KE Parker M Clarke C Stenhouse G. 2004. Scat detection dogs in wildlife research and management: application to grizzly and black bears in the Yellowhead Ecosystem, Alberta, Canada. Can J Zool. 82:474–492

[CIT0038] Wachowiak M. 2011. All in a sniff: olfaction as a model for active sensing. Neuron. 71:962–973 21943596 10.1016/j.neuron.2011.08.030 PMC3237116

[CIT0039] Weber CM Cauchi M Patel M Bessant C Turner C Britton LE Willis CM. 2011. Evaluation of a gas sensor array and pattern recognition for the identification of bladder cancer from urine headspace. Analyst. 136:359–364 20967397 10.1039/c0an00382d

[CIT0040] Wesson DW Carey RM Verhagen JV Wachowiak M. 2008. Rapid encoding and perception of novel odors in the rat. PLoS Biol. 6:e82 18399719 10.1371/journal.pbio.0060082 PMC2288628

[CIT0041] Wesson DW Verhagen JV Wachowiak M. 2009. Why sniff fast? The relationship between sniff frequency, odor discrimination, and receptor neuron activation in the rat. J Neurophysiol. 101:1089–1102 19052108 10.1152/jn.90981.2008 PMC2657070

[CIT0042] Zhang L Tian F Dang L Li G Peng X Yin X Liu S. 2013. A novel background interferences elimination method in electronic nose using pattern recognition. Sensor Actuat A-Phys. 201:254–263

